# Saccades during Attempted Fixation in Parkinsonian Disorders and Recessive Ataxia: From Microsaccades to Square-Wave Jerks

**DOI:** 10.1371/journal.pone.0058535

**Published:** 2013-03-13

**Authors:** Jorge Otero-Millan, Rosalyn Schneider, R. John Leigh, Stephen L. Macknik, Susana Martinez-Conde

**Affiliations:** 1 Department of Neurobiology, Barrow Neurological Institute, Phoenix, Arizona, United States of America; 2 Department of Signal Theory and Communications, University of Vigo, Vigo, Spain; 3 Veterans Affairs Medical Center, Case Western Reserve University, Cleveland, Ohio, United States of America; 4 Department of Neurosurgery, Barrow Neurological Institute, Phoenix, Arizona, United States of America; University of California Davis, United States of America

## Abstract

During attempted visual fixation, saccades of a range of sizes occur. These “fixational saccades” include microsaccades, which are not apparent in regular clinical tests, and “saccadic intrusions”, predominantly horizontal saccades that interrupt accurate fixation. Square-wave jerks (SWJs), the most common type of saccadic intrusion, consist of an initial saccade away from the target followed, after a short delay, by a “return saccade” that brings the eye back onto target. SWJs are present in most human subjects, but are prominent by their increased frequency and size in certain parkinsonian disorders and in recessive, hereditary spinocerebellar ataxias. Here we asked whether fixational saccades showed distinctive features in various parkinsonian disorders and in recessive ataxia. Although some saccadic properties differed between patient groups, in all conditions larger saccades were more likely to form SWJs, and the intervals between the first and second saccade of SWJs were similar. These findings support the proposal of a common oculomotor mechanism that generates all fixational saccades, including microsaccades and SWJs. The same mechanism also explains how the return saccade in SWJs is triggered by the position error that occurs when the first saccadic component is large, both in the healthy brain and in neurological disease.

## Introduction

During attempted visual fixation of a stationary target, saccadic intrusions (predominantly horizontal saccades that “intrude on” or interrupt accurate fixation) and fixational eye movements (including microsaccades, drift and tremor) continuously change the position of gaze [1]–[3]. Microsaccades, which counteract perceptual fading resulting from sensory adaptation [4]–[8], are too small (often <0.5 deg) to be evident during clinical examination. Square-wave jerks (SWJs), a type of saccadic intrusion consisting of a small saccade away from the fixation target, followed by a corrective saccade back towards the target, can be large enough to be evident clinically. SWJs occur in normal, healthy humans [1], [9], but are a clinically prominent feature–due to increased frequency and magnitude–in a number of neurological diseases, especially parkinsonian disorders [9] and recessive spinocerebellar ataxias [10].

We recently studied microsaccades and SWJs in healthy controls and patients with progressive supranuclear palsy (PSP), a parkinsonian disorder in which SWJs are a feature of the clinical syndrome [11], [12]. Microsaccade magnitude was correlated with SWJ coupling in both healthy subjects and PSP patients; that is, larger microsaccades were more likely to trigger return saccades, and thus form SWJs, than smaller microsaccades. These findings, taken with prior studies, are consistent with the idea that saccadic intrusions and microsaccades are essentially the same type of eye movement [12], [13]. Moreover, our data indicated that a common mechanism can account for the generation of microsaccades and SWJs, and explained how the position error following from a large first saccade could trigger the return saccade in SWJs, both in the healthy brain and in PSP [14]. We also found that microsaccades and SWJs in PSP were slow and had a reduced vertical component, consistent with the vertical saccadic palsy that distinguishes PSP patients from those with other parkinsonian-dementia syndromes, and may result from damage to the midbrain regions that produce vertical saccades [15]. Midbrain involvement appears to be distinct from the mechanism that leads to frequent SWJs, and may reflect impaired inhibition of the superior colliculus by basal ganglionic circuits, especially substantia nigra pars reticulata (SNpr) [16].

Here we studied representative patients with other disorders characterized by clinically evident saccadic intrusions, including idiopathic Parkinson’s disease (PD), multiple system atrophy (MSA), corticobasal syndrome (CBS), and a form of recessive spinocerebellar ataxia. Our goals were: (1) to determine if a common oculomotor mechanism [14] might explain the generation of microsaccades and saccadic intrusions (particularly SWJs) over a broad range of movement disorders, and (2) to identify distinguishing features of fixational saccades that might aid correct diagnosis of each condition. From here on, for simplicity, we will refer to all saccades made during attempted fixation, regardless of size, as fixational saccades or, simply, saccades.

## Materials and Methods

### Subjects

Five subject groups participated in the experiments: The PSP group comprised 10 patients (5 females, age range 58–74, median 66.5 years) diagnosed as probable PSP according to the criteria of the NINDS-SPSP study [17]. The PD group comprised 4 patients (1 female, age range 67–80) scaled in the Hoehn-Yahr Scale for Parkinsons’ disease [18]. The CBS group comprised 2 patients (1 female, ages 71 and 79), diagnosed in accord with the criteria of Boeve and colleagues [19]. The MSA group comprised 2 patients (1 female, ages 68 and 71), diagnosed in accord with the criteria of Gilman and colleagues [20].The recessive ataxia group comprised two brothers (ages 57 and 61) with spinocerebellar ataxia with saccadic intrusions (SCASI – [21]). The Control group comprised 14 subjects: 7 age-matched healthy subjects (1 female, age range 58–74, median 65 years, visual acuity better than 20/30 ) and 7 younger controls (5 females, age range 22–38 years, median 31 years, normal or corrected-to-normal vision). Data from the PSP and Control groups were reported in [12], [22]. We previously established that age-matched and younger controls had equivalent gaze dynamics (i.e. saccadic magnitude and direction distributions) [12]; thus in the present study we considered all control subjects together. **Table 1** summarizes the clinical features of all participating patients. The patient groups and the aged-matched controls were tested at the Veterans Affairs Medical Center, Case Western Reserve University (Cleveland OH); each subject participated in one experimental session lasting approximately 30 minutes. The younger controls were tested at the Barrow Neurological Institute (Phoenix, AZ); each subject participated in 3 experimental sessions, of about 60 minutes. All subjects were naive to the purpose of the experiments, had the capacity to consent, and gave written informed consent in accordance with the Cleveland Veterans Affairs Medical Center Institutional Review Board or the St. Joseph’s Hospital and Medical Center Institutional Review Board, and with the Declaration of Helsinki. No research was conducted outside of the country of residence of the authors (USA).

**Table 1:** Clinical features of all patients.

Subject/Sex/Age	Disease duration	Clinical Features/Ocular Motor Abnormalities*	CNS Medications	Date of record
PD1/F/73	3 yr	HYS 3/Saccades - normal speed, mild hypometria	Levodopa/carbidopa	1/9/2003
PD2/M/80	2 yr	HYS 3/Saccades - normal speed, mild hypometria	Levodopa/carbidopa	1/8/2002
PD3/M/67	6 yr	HYS 3/Saccades –normal speed, mild hypometria	Levodopa/carbidopa	2000
PD4/M/68	13 yr	HYS 2/Saccades –normal speed, mild hypometria	Levodopa/carbidopa, entacapone, pramipexole	10/26/11
CBGD1/F/79	2 yr	Apraxia, rigidity/Saccades –normal speed, mildly hypometric but increased latency	Levodopa/carbidopa	2000
CBGD2/M/71	3 yr	Apraxia, alien limb, rigidity/Saccades –normal speed, mildly hypometric but increased latency	Levodopa/carbidopa	2000
MSA171/F/71	4 yr	Asymmetric akinetic-rigid without tremor; abnormal autonomic functions/Saccades –normal speed, mild hypometria	None	2000
MSA2/M/68	4 yr	Asymmetric akinetic-rigid with mild tremor; abnormal autonomic function/Saccades –normal speed, mild hypometria	Oxybutinin	2000
SCA7/M/69	8 yr	Limb and gait ataxia/Saccades –slow and hypermetric	None	02/09/11
SCASI/M/61	20 yr	Limb and gait ataxia; neuropathy/Saccades –normal speed, hypermetric; large saccadic intrusions during fixation	Clonazepam, fluoxetine,memantine	02/10/11
PSP01/M/61	4	Falls/Vertical gaze palsy; saccades – slow downward	None	4/10/07
PSP02/M/74	6	Slow speech, loss of balance/Saccades – slow vertically	None	4/11/07
PSP03/F/61	4	Loss of balance/Saccades – slow vertically	Levodopa	4/3/07
PSP04/F/65	3	Dysarthria, fixed stare, falls/Saccades – small, slow and difficult to initiate vertically	None	2/13/07
PSP05/M/58	5	Falls/Vertical gaze palsy; saccades – slow and small vertically	Paroxetine	2/27/07
PSP06/F/66	4	Dizziness, falls, dysphagia/Vertical gaze palsy;saccades – slow, small and difficult toinitiate vertically	None	5/30/07
PSP07/F/67	5	Falls, dysphagia/Saccades – slow, small and difficult to initiate vertically	Donepezil, memantine,levodopa/carbidopa	7/10/07
PSP08/M/67	3	Falls, dysphagia/Saccades – slow, small and difficult to initiate vertically	Fluoxetine, Levodopa/carbidopa, donepezil	7/17/07
PSP09/M/70	7	Falls, dysphagia/Downward gaze palsy; saccades – slow and small downward	Levodopa/carbidopa	6/12/07
PSP10/F/72	2	Falls/Vertical gaze paresis; saccades – slow vertically	Levodopa/carbidopa	5/22/07

* On clinical examination, most patients and age-matched controlled subjects showed mild limitation of upgaze and mild impairment of convergence and smooth pursuit. HYS: Hoehn-Yahr Scale for Parkinsons’ disease [18] **All PSP patients showed impaired smooth pursuit and vergence eye movements.

### Recordings

For patients and age-matched controls, eye movements were recorded monocularly (5 Controls, 4 PSP, 2 PD, 2 SCASI) or binocularly (1 Control, 6 PSP, 2 PD, 2 CBS, 2 MSA) with the magnetic field/search coil technique [23]; search coils were calibrated on a protractor device prior to each experimental session. Subjects sat in a vestibular chair with their heads stabilized by a chair-fixed restraint. Coil signals were low-pass filtered (bandwidth 0–150 Hz) prior to digitization at 500 Hz with 16-bit precision, as described previously [24]. The standard deviation of the noise of the coil system was 0.02 deg. The younger controls rested their head on a chin-rest while their eye position was acquired non-invasively with a fast video-based eye movement monitor (EyeLink II, SR Research, Ontario, Canada). The EyeLink II system records eye movements simultaneously in both eyes (temporal resolution 500 samples/s; instrument noise 0.01 deg RMS).

Absolute eye position measurements are less reliable than eye-position change measurements in all eye tracking systems, especially in the case of video trackers. Both magnetic/search coil and video-tracking techniques detect small saccades reliably, however, because saccade detection depends on relative changes in eye position, rather than on the absolute position of the eye. To quantify absolute eye position errors we used an estimated reference center position rather than the zero position obtained from the eye-tracking system’s calibration protocol. Thus, to calculate the gaze position error at the end of each saccade, we estimated the distance between a given gaze position and the median gaze position during that trial**.**

### Experimental Design

Patients and age-matched controls were asked to maintain gaze fixation during the recordings (minimum fixation duration: 10 s; maximum fixation duration: 120 s). Subjects viewed a small target (laser spot subtending 0.1°) placed at central position on a tangent screen at 1.4 m in an otherwise dark room. Verbal encouragement was provided to subjects to sustain steady fixation of the small target during the test period, allowing occasional blinks. Five subjects (1 PD, 2 MSA and 2 CBS subjects) performed a vergence task; here we analyzed only the periods of stable fixation (minimum duration: 800 ms). The younger controls fixated a red cross (0.75 degrees wide) within a 2 deg x 2 deg window, on a 50% gray background. The cross was presented on the center of a linearized video monitor, at 57 cm. The subjects received auditory feedback (a short beep) whenever their gaze left the fixation window for more than 500 ms (<500 ms gaze excursions were permitted to allow for blinks). Eye movements exceeding the fixation window were also recorded.

### Saccade Detection

We identified all saccadic eye movements automatically with an objective detection algorithm (see [25], for details). This method detects saccades in the two-dimensional velocity space using a threshold that adapts to the level of noise of each recording. For subjects in whom eye position was recorded binocularly, we first detected the saccades from each eye’s recording and then reduced the amount of potential noise [26] by analyzing only binocular saccades, that is, saccades with a minimum overlap of one data sample in both eyes [12], [27].

Some saccades are followed by a fast and small saccadic eye movement in the opposite direction, called dynamic overshoot, which is often more prominent in the eye that moves in the abducting direction [28]. Unlike the return saccade in a SWJ, a dynamic overshoot follows a saccade without latency between the two movements. We identified dynamic overshoots as saccades that occurred less than 20 ms after a preceding saccade [7], [29]–[31], and considered them part of the preceding saccade (i.e. we did not regard them as new saccades). That is, we discarded the second saccade and modified the end point of the first saccade to include the overshoot.

To calculate the saccadic duration/magnitude relationship [32]–[34], we combined eye velocity and acceleration measures to optimize our estimation of saccadic duration. We detected the beginning of a microsaccade as the last sample before the point of maximum velocity where acceleration changed sign from negative to positive, and velocity was below 20 deg/s. We detected the end of a microsaccade as the first sample after the point of maximum velocity where acceleration changed sign from negative to positive, and velocity was below 20 deg/s.

### SWJ Detection

**Figure 1:**
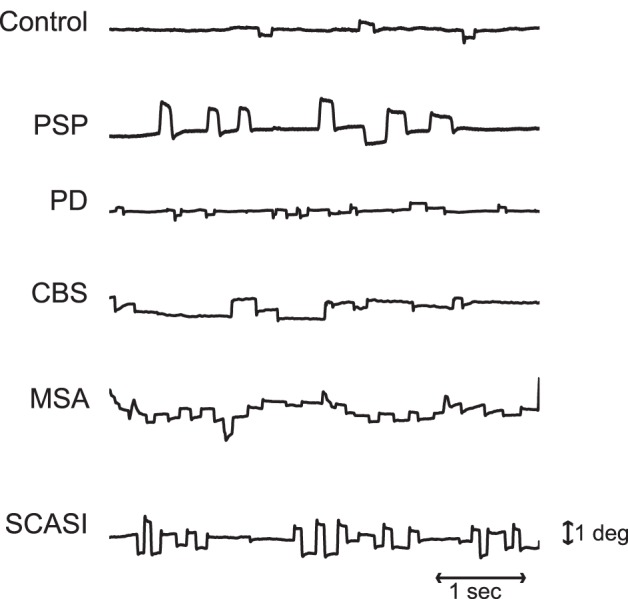
Examples of saccadic intrusions in a control subject, a PSP patient, a PD patient, a CBS patient, a MSA patient and a SCASI patient. SWJs were present in all subject groups, although they were smaller and less frequent in healthy controls. Each trace represents a 5 s recording of horizontal eye positions containing SWJs. Horizontal position and timescales for all traces are as in the bottom trace.

We defined a SWJ as the combination of one small saccade that moves the eye away from the fixation target, followed after a short period by a second corrective saccade directed back towards the target [1], [3], [14], [35] (**Figure 1**). To characterize SWJs in an objective manner, we first identified all individual saccades up to 5 degrees [12]. We chose this 5-degree upper magnitude threshold to include the range of SWJ magnitudes reported previously in healthy subjects (0.1–4.1 deg; [1], and to allow for potentially larger SWJs magnitudes in patients [12].

We identified SWJs using the algorithm developed in [12]. This method measures the similarity between a given saccade pair (that is, a pair of consecutive saccades) and ideal SWJ. In an “ideal SWJ” the two saccades are separated by a short interval (i.e. 200 ms), have the same magnitudes, and their directions are exactly opposite [12]. We calculated an SWJ index based on the three defining SWJ characteristics described above: a) the direction dissimilarity of first and second saccade, b) the magnitude similarity of first and second saccade, and c) the temporal proximity of first and second saccade. The SWJ index provides a single, continuous variable between zero and one for each saccade pair. Values closer to one indicate more similarity to the ideal SWJ. If a saccade pair’s SWJ index was larger than a given threshold [12], we classified the pair as a potential SWJ.

### Statistics

Previous to testing the potential differences between pairs of subject groups, we tested the main effect using one-way ANOVA. To correct for multiple comparisons we used the Tukey Honest Differences (HSD) method. We also conducted the Linear Discriminant Analysis [36], which uses a linear combination of a set of variables to classify different samples into multiple groups. To measure the ability of the variables to assign patients to the correct diagnosis categories, we used a Leave One Out cross-validation method [37]. That is, for each given sample, we trained the model with all the samples except for that particular one, and then asked whether that sample was classified correctly.

The parametric relationship between saccadic peak velocity and saccadic magnitude [32] is approximately linear for small saccades. Thus, we used a simple linear regression to compare the slopes of the peak velocity-magnitude relationship across subject groups.

## Results

### A Common SWJ Coupling Mechanism in the Intact Brain and in Neurological Disease

SWJs are prevalent in numerous neurological disorders, including those studied here, and they are common in healthy subjects as well [1], [3], [12]. We proposed previously that PSP patients and healthy controls share a common square-wave coupling mechanism [12], [14]. Here, we set out to determine if this coupling mechanism might also apply to PD, to other parkinsonian disorders, and to recessive ataxia: if so, it would be an important piece of evidence supporting our proposal that a common saccade generation mechanism can explain some of the oculomotor deficits associated with each of these diseases.

**Figure 2:**
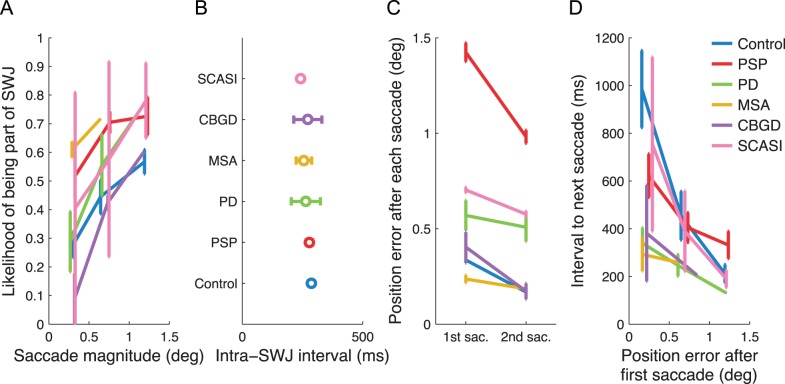
A common square-wave coupling mechanism. A) Correlation between saccade size and likelihood of being part of a SWJ. B) Intra-SWJ intervals across groups. C) Position error (see Methods for details) at the end of the first versus second saccade in a SWJ. D) Relationship between the estimated position error at the end of each saccade and the inter-saccadic interval to the next saccade. Error bars in all panels represent the standard error of the mean across subjects.

We found that in all patients – irrespective of their diagnosis – larger saccades were more likely to be part of SWJs (logistic regression, p<0.05, see **Figure 2A**). Thus, the present results extend the correlation between saccade size and SWJ coupling, previously found in PSP patients and healthy controls [12], to all subject groups, including PD, MSA, CBS and SCASI patients.

If all subject groups share the same SWJ-coupling mechanism, as suggested by the data above, it follows that they should have equivalent intra-SWJ intervals (i.e. the time it takes to trigger the second saccade should be very similar across groups). Our analysis confirmed this hypothesis (one-way ANOVA; p>0.05, see also **Figure 2B**), consistent with the existence of a common SWJ coupling mechanism in the intact brain and in neurological disease.

In all subject groups, return SWJ saccades reduced the eye position errors introduced by the initial saccades (t-test; p<0.05 in all groups except for PD, which showed the same trend, see **Figure 2C**). (Note that our SWJ detection algorithm did not take into account the eye position error at the beginning or end of the saccades, and so it did not require this error reduction (see Materials and Methods)). Moreover, the latencies of the return SWJ saccades were inversely correlated to eye position errors after the first SWJ saccade (p<0.05 in all groups, see **Figure 2D**). This finding supports the hypothesis that gaze position errors trigger corrective saccades during attempted fixation [38], and is consistent with the proposal of a common SWJ coupling mechanism in healthy subjects and in patients [14].

### Distinctive Properties of Fixational Saccades in PD Versus PSP

Previous research showed that fixational saccades are larger, slower, more frequent and more horizontal in PSP patients than in healthy controls [12], [39]. Here we aimed to identify any fixational saccade properties that could distinguish PD from PSP patients.

**Figure 3:**
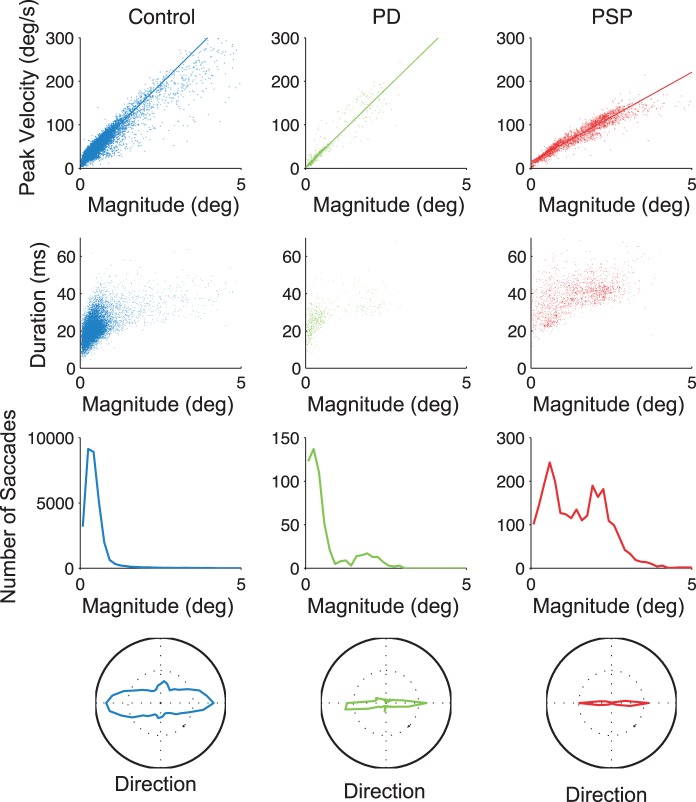
Characteristics of fixational saccades across subject groups. First row, saccadic peak velocity/magnitude relationships. Second row, saccadic duration/magnitude relationships. Third row, saccade magnitude distributions. Fourth row, polar histograms of saccade directions. Each graph shows the combined data for all subjects in each group.

To discriminate between groups (PD, PSP, and controls), we focused on four saccadic properties likely to reflect the differential involvement of brain areas related to the oculomotor system: saccade rate, saccade magnitude, peak velocity-magnitude relationship slope (see Materials and Methods), and vertical component of saccade direction (**Figures 3** **,** **4**).

**Figure 4:**
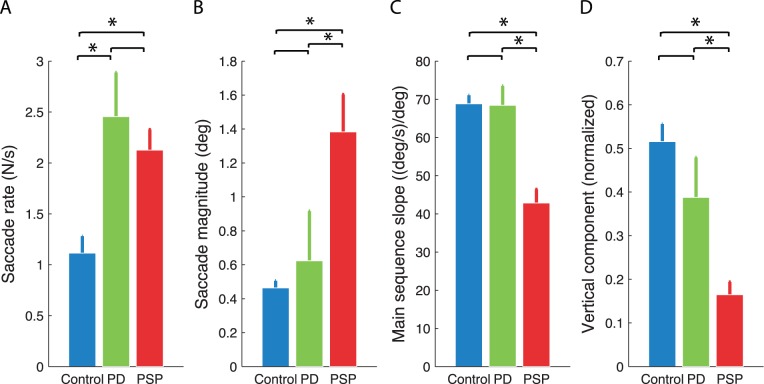
Saccadic parameters in PD patients, PSP patients and healthy controls. Saccade rates, magnitudes, peak velocity-magnitude relationship slopes and vertical components (of saccade direction) are indicated. Bars represent the average value across subjects of each group and the error bars indicate the standard error of the mean. Asterisks show significance (p<0.05, t-test).

Statistical analyses showed that the three groups (PD, PSP, and controls) were statistically distinct from one another (main effect one-way ANOVA) for saccade rate (p = 0.005), saccade magnitude (p = 0.000001), slope of the peak velocity-magnitude relationship (p = 0.000001) and normalized saccade vertical component (p = 0.00004). Pair-wise comparisons revealed differences between the PD, PSP and control subjects. See **Figure 4** **and** **Table 2**.

**Table 2:** Saccadic parameters in PD patients, PSP patients and healthy controls.

Saccade rate (ANOVA, p = .005)		Tukey HSD test p-value		
	Average (N/s)	PSP	PD	Control
PSP (N = 10)	2.1±0.2	−	0.9	**0.001**
PD (N = 4)	2.2±0.3	0.9	−	**0.008**
Control (N = 14)	1.1±0.2	**0.001**	**0.008**	−
**Saccade magnitude (ANOVA, p = .00000)**		**Tukey HSD test p-value**		
	**Average (deg)**	**PSP**	**PD**	**Control**
PSP (N = 10)	1.38±0.22	−	**0.03**	**0.0002**
PD (N = 4)	0.62±0.3	**0.03**	−	0.9
Control (N = 14)	0.46±0.04	**0.0002**	0.9	−
**Peak velocity-magnitude relationship slope (ANOVA, p = .00000)**		**Tukey HSD test p-value**		
	**Average ((Deg/s)/s)**	**PSP**	**PD**	**Control**
PSP (N = 10)	43±4	−	**0.0001**	**0.000001**
PD (N = 4)	71±4	**0.0001**	−	0.9
Control (N = 14)	69±2	**0.000001**	0.9	−
**Vertical component (ANOVA, p = .00004)**		**Tukey HSD test p-value**		
	**Average (normalized)**	**PSP**	**PD**	**Control**
PSP (N = 10)	0.16±0.03	−	**0.01**	**0.000002**
PD (N = 4)	0.40±0.07	**0.01**	−	0.3
Control (N = 14)	0.51±0.04	**0.000002**	0.3	−

Bold text indicates statistical significance.

PD patients had higher saccadic frequencies than controls. Other saccadic properties (including saccadic magnitude, velocity and direction) were unaffected and thus did not differ from those in control subjects (**Figures 3** **,** **4**).

PSP patients had more frequent, larger, more horizontal, and slower saccades than controls (Otero-Millan et al., 2011) (**Figures 3** **,** **4**). Slow PSP saccades had correspondingly long durations (**Figure 3** **, second row**).

Saccadic frequency was comparable in PD and PSP (**Figure 4A**). Saccadic dynamics that distinguished PD from PSP included saccadic direction (the vertical component was larger in PD than in PSP; **Figure 3** **, fourth row,** **Figure 4D**), saccadic magnitude (smaller in PD than in PSP; **Figure 3** **, third row,** **Figure 4B**), and saccadic velocity (higher in PD than in PSP; **Figure 3** **, first row,** **Figure 4C**).

Thus, a patient exhibiting a decreased vertical saccadic component will likely suffer from PSP, whereas a patient with high-frequency saccades of normal direction, magnitude, and speed, will probably suffer from PD.

To quantify to what extent saccade rate, saccade magnitude, peak velocity-magnitude relationship slope, and vertical component could differentiate between the PD, PSP and control groups, we performed a Linear Discriminant Analysis. Seventy nine per cent of the subjects were assigned to the correct diagnosis group, using the Leave One Out cross-validation method (see Materials and Methods for details).

## Discussion

We set out to compare the properties of fixational saccades ranging from microsaccades to SWJs in healthy subjects and representative patients with several disorders known to show prominent saccadic intrusions. Disruption of steady fixation by saccadic intrusions (i.e. SWJs) is often clinically evident in patients with the parkinsonian-dementia spectrum of disorders and recessive spinocerebellar ataxia such as Friedreich’s ataxia [10], [21]. So impressive are these saccadic intrusions in some such patients that attempts have been made to use their frequency and size to aid diagnosis [9]. One problem with this approach is that normal, healthy subjects can show frequent saccadic intrusions as well. Thus, Pinnock and colleagues found some overlap between PSP, MSA, PD, and control subjects [39]. Could other features of saccadic intrusions, such as their speed and direction, aid diagnosis? In a prior study, we found that fixational saccades in PSP patients were slower and had a smaller vertical component than in control subjects, consistent with the distinctive deficits of saccade dynamics in PSP [12]. Here we found that some of these properties also help to distinguish PSP from PD patients: fixational saccades were slower, larger, and had a smaller vertical component in PSP than in PD. Saccadic rates were equivalent in PD and PSP, and thus higher than in control subjects. Differences between fixational saccades made by patients with other diagnoses were modest. However, our main finding is that square wave coupling was similar in all patient groups and control subjects: larger saccades were more likely to be part of SWJs, and the interval between the first and second saccade of SWJs remained similar across subject categories.

Thus, our results indicate that SWJ coupling is similar in healthy controls and in all patient groups, and suggest that a common saccade generation mechanism can explain the oculomotor features that characterize each disorder. In order to interpret these findings, we first discuss the mechanisms that trigger saccades during attempted fixation, and then apply this scheme to examine the possible pathophysiology of saccadic intrusions encountered in the neurological disorders studied here.

### Generation of Saccadic Intrusions and Microsaccades

Activity in the superior colliculus (SC) triggers fixational saccades, both in response to spontaneous neural fluctuations and to fixation error signals [14], [40], [41]. Thus, in a SWJ, random fluctuations in neural activity may drive the first saccade, producing a fixation error which in turn causes the second (corrective) saccade. This is consistent with research showing that SC activity encodes gaze position errors and is related to saccade initiation [42], [43].

**Figure 5:**
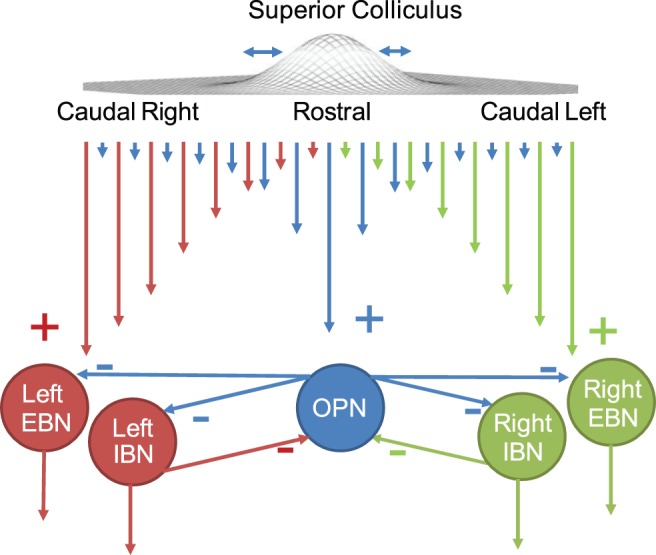
Microsaccade triggering model [**14**]. SC neurons present two gradients of connectivity, one that is strongest between rostral SC and OPNs, and one that is strongest between caudal SC and EBNs and IBNs [59]–[61] (longer lines represent stronger connections). The mutually inhibited OPNs and IBNs act as a trigger. During fixation, rostral SC activity drives the OPNs that inhibit the EBNs and IBNs. Directly preceding the launch of a microsaccade, activity in the rostral area shifts slightly caudally. At some point the balance of inhibition is broken, and the IBNs inhibit the OPNs more than the OPNs inhibit the IBNs. Then the EBNs start to burst initiating the microsaccade. Note that this representation is a one-dimension simplification of the circuit. The circuit functions in the same manner for vertical (up and down) BNs. Minus signs indicate inhibitory connections, plus signs excitatory ones.

We proposed previously [14] that the microsaccade-triggering circuit is formed by the SC and two mutually-inhibiting groups of neurons in the brainstem: excitatory and inhibitory burst neurons (EBNs and IBNs [44]) in the reticular formation and omnipause neurons (OPNs [45]) in the raphe. During fixation, the OPNs are tonically active and keep EBNs and IBNs inhibited [46]. Due to spontaneous activity and/or fixation error, the activity of the SC map moves away from the location that represents the center of gaze (i.e. rostral SC), increasing the input to the BNs and decreasing the input to the OPNs. At some point, the input to the IBNs becomes high enough to overcome the decreased inhibition coming from the OPNs [47]. This results in a small burst of activity in the EBNs, triggering a microsaccade (**Figure 5**).

Several cortical areas, including the frontal eye fields, supplementary eye fields, and dorsolateral prefrontal cortex, drive the SC. Although direct projections from frontal areas to the SC do exist, indirect projections via the basal ganglia seem important; these reach the SC via SNpr, which acts as an inhibitory gateway. Parallel pathways from the frontal cortex pass via the caudate, external segment of globus pallidus, and subthalamic nuclei, with some connections contributing more to initiation, and others to suppression, of saccades [16]. How might these pathways be compromised in our patients?

### Pathophysiology of Saccadic Intrusions in Neurological Disease

Each of the parkinsonian disorders studied here involves the basal ganglia. Impaired basal ganglia function due to disease might unbalance the normal governance of saccade initiation and suppression, mediated by the parallel basal ganglionic pathways to the SC [16]. In PD, the damage is concentrated in the dopaminergic portion of substantia nigra, which modulates the activity of caudate neurons. Thus, in PD, there is impaired ability to generate self-paced saccades but also to suppress unwanted saccades [48], [49]. Perhaps the best direct support for this view is that acute, therapeutic lesions of the globus pallidus (pallidotomy) for severe PD increase the frequency of SWJs [50], [51]. Direct or indirect impairment of SNpr function will compromise the inhibitory control of the SNpr in the SC, which may lead to increased neural fluctuations. Increased fluctuations in SC activity would have the effect of raising the rates of fixational saccades, consistent with the present results (**Figures 4A**). However, because the brainstem and the cerebellum are relatively spared in PD, fixational saccades should have normal parameters, such as magnitude, velocity or direction, as observed here.

In PSP, the characteristically slow saccades (**Figure 3** **, first row,** **Figure 4C**) indicate neural damage to BNs in the brainstem (especially BNs in the rostral interstitial nucleus of medial longitudinal fasciculus (riMLF), which controls vertical gaze) [15], [52]. Damage to BNs might also explain why PSP patients, but not PD patients, produce increased magnitude saccades during attempted fixation. If one subpopulation of BNs is impaired (i.e. vertical BNs in PSP), only more caudal fluctuations in SC activity will produce enough drive for the remaining healthy BNs to overcome the inhibition from the OPNs and trigger saccades. Saccades triggered in this fashion will be larger than normal. Some patients with CBS and MSA may show slowing of vertical saccades as well, and recent evidence suggests that this could be a consequence of midbrain damage [15].

In recessive ataxias, saccadic intrusions are often prominent, but these patients do not show parkinsonian features. In their case, impaired control of BNs by the fastigial nucleus may be the culprit; such individuals usually show saccadic hypermetria, an impressive finding following fastigial nucleus inactivation [53]–[55]. According to our hypothesis, the second saccade in SWJs is triggered to correct the gaze position error produced by the first saccade. A hypermetric second saccade would also produce error, thus triggering another saccade. Therefore, impaired output from the fastigial nucleus would lead to more frequent SWJs. In the case of our SCASI patients, their SWJs indeed showed “overshoot” so that their eyes did not return to the fixation point, but instead oscillated around it– a type of saccadic intrusion called “macrosaccadic oscillations” [56].

In conclusion, we have found evidence to support our proposal that microsaccades and saccadic intrusions, such as SWJs, form a continuum, and show similarities in both healthy subjects and in patients with a variety of neurological disorders. We propose that the initial saccade depends on neural noise in the superior colliculus and brainstem saccade-generating circuits, both in health and in neurological disease. In health, microsaccades aid vision by counteracting adaptation [4], [7], [8]. With disease affecting basal ganglion circuits, inhibitory control is released from the SC, promoting saccadic intrusions. The probability of a return saccade following a saccadic intrusion is a function of the size of the initial movement; in disease states, especially those affecting the cerebellum, the characteristics of this return saccade may be affected. Because SWJs share a common neural circuit with saccades, they will likely show pathological characteristics related to larger voluntary saccades. Future research may build on the current results to provide further insights into the pathogenesis of parkinsonian disorders, and to develop accessible approaches by which eye movements can be used to evaluate treatments and enhance early diagnosis.

A remaining issue is why human microsaccades and saccadic intrusions are predominantly horizontal, especially as microsaccades in macaques do not typically show a horizontal preference [57], [58]. Future studies should investigate the prevalence of primate SWJs [57] and their relationship with microsaccade direction.
